# Biodegradable Antibacterial Nanostructured Coatings on Polypropylene Substrates for Reduction in Hospital Infections from High-Touch Surfaces

**DOI:** 10.3390/nano16020080

**Published:** 2026-01-06

**Authors:** Mariamelia Stanzione, Ilaria Improta, Maria Grazia Raucci, Alessandra Soriente, Marino Lavorgna, Giovanna Giuliana Buonocore, Roberto Spogli, Anna Maria Marcelloni, Anna Rita Proietto, Ilaria Amori, Antonella Mansi

**Affiliations:** 1 Institute of Polymers, Composites and Biomaterials, National Research Council, P.le E. Fermi 1, 80055 Portici, Italy; mariamelia.stanzione@cnr.it (M.S.); ilariaimprota@cnr.it (I.I.); mariagrazia.raucci@cnr.it (M.G.R.); alessandra.soriente@cnr.it (A.S.); marino.lavorgna@cnr.it (M.L.); 2 Prolabin & Tefarm Srl, Via Dell’Acciaio 9, 06136 Perugia, Italy; roberto.spogli@prolabintefarm.com; 3 Department of Occupational and Environmental Medicine, Epidemiology and Hygiene, Italian Workers’ Compensation Authority (INAIL), Via Fontana Candida 1, Monte Porzio Catone, 00078 Rome, Italy; a.marcelloni@inail.it (A.M.M.); a.proietto@inail.it (A.R.P.); i.amori@inail.it (I.A.); a.mansi@inail.it (A.M.)

**Keywords:** antimicrobial polymeric nanomaterials, active coatings, pathogen transmission, hospital-acquired infections

## Abstract

Healthcare-associated infections (HCAIs) remain a significant global challenge, as pathogenic microorganisms can persist on hospital surfaces and medical equipment, contributing to severe infections and epidemic outbreaks. Conventional preventive measures, including disinfection procedures and personal protective equipment, are often insufficient to ensure complete microbial control, prompting interest in innovative antimicrobial surface technologies. This study reports the design, preparation, and comprehensive characterization of chitosan- and poly(ε-caprolactone)-based antibacterial coatings incorporating chlorhexidine-loaded zirconium phosphate (ZrPCHX) nanoparticles. Coatings were deposited by optimized spray and brush techniques to obtain uniform, adherent, and well-defined films. Their morphological, physicochemical, mechanical, and cytocompatibility properties were systematically evaluated, and antibacterial efficacy was assessed against clinically relevant pathogens following ISO 22196:2011 and additional protocols simulating realistic hospital conditions. Both coating systems demonstrated pronounced antibacterial activity, with the PCL-based formulation exhibiting a faster and broader bactericidal effect while maintaining good cytocompatibility. These findings support the potential of the developed nanostructured coatings as sustainable and scalable materials for the active decontamination of high-touch hospital surfaces, offering continuous antimicrobial protection and contributing to a reduction in HCAI incidence.

## 1. Introduction

Hospital-acquired infections (HAIs) represent a persistent and critical challenge for healthcare systems worldwide, contributing to increased morbidity, prolonged hospitalization, and substantial healthcare costs [1]. High-touch hospital surfaces (e.g., bed rails, door handles, switches, medical carts) play a pivotal role in pathogen transmission, including multidrug-resistant strains like *Staphylococcus aureus* (MRSA), *Klebsiella pneumoniae*, and *Acinetobacter baumannii* [2,3,4]. Although routine disinfection and cleaning protocols are indispensable, their antimicrobial protection is short-lived and often insufficient to eliminate pathogens, especially in complex hospital environments [5]. Therefore, there is a growing need for preventive strategies capable of ensuring continuous antimicrobial protection directly at the contact surface [6].

In this context, nanostructured antibacterial coatings, especially ones derived from biodegradable polymers, have emerged as a promising solution to mitigate microbial contamination on healthcare surfaces [7,8]. These functional nanocoatings can be engineered to establish both physical and chemical interactions with the underlying substrates, thereby inhibiting or delaying microbial colonization while minimizing the environmental impact at end of life [9].

Several antibacterial coatings have already been reported in the literature and, in some cases, successfully commercialized. Most of them are based on inorganic nanofillers such as silver, copper, or zinc oxide nanoparticles, embedded into polymeric or sol–gel matrices, which confer a broad-spectrum antimicrobial effect [10].

Various antimicrobial surface treatments are currently available for commercial use, mostly based on silver [11,12] or copper [13,14], and are designed for healthcare applications. These treatments are incorporated into polymers and paints. However, such products often suffer from poor biodegradability and are difficult to remove, and their antimicrobial action can decline significantly after repeated cleaning cycles. This highlights the need for innovative nanocoatings that combine strong substrate adhesion, long-lasting antibacterial efficacy, and controlled renewal of the functional surface while remaining environmentally sustainable [15,16].

The innovative aspect of this work lies in the incorporation of the active compound into a nanostructured system subsequently embedded into a biodegradable polymer, designed to achieve a rapid yet long-lasting bactericidal effect. By exploiting the nanostructured architecture, it is possible to enhance the stability of the active agent and promote a rapid onset of antimicrobial activity, potentially supporting its efficacy over extended periods of use. In line with this strategy, the incorporation of functional nanofillers into biodegradable matrices has increasingly been adopted to enhance performance and confer prolonged antibacterial activity [17,18]. Zirconium phosphate intercalated with chlorhexidine (ZrPCHX) was embedded within carboxymethylcellulose hydrocolloid films intended for wound dressings, providing sustained release of chlorhexidine, strong antibiofilm activity, and reduced cytotoxicity [19]. A different delivery system based on novel calcium hydroxide [Ca(OH)_2_] microparticles loaded with chlorhexidine (CHX) was studied by Priyadarshini et al., designed for potential dental therapeutic and preventive applications [20]. The relationship between formulation parameters, structural features, and final material properties is critical for designing effective antimicrobial coatings, as it directly influences the functional performance and overall efficiency of the coating.

Among various strategies, the development of antibacterial coatings utilizing biodegradable and biocompatible polymeric matrices has garnered attention. Chitosan (CS) and poly(ε-caprolactone) (PCL) are among the most widely investigated biodegradable polymers due to their excellent film-forming capability, biocompatibility, and mechanical versatility [21,22,23]. Chitosan, a cationic polysaccharide derived from chitin, is well known for its intrinsic antimicrobial activity, high surface reactivity, and ability to form stable hydrogels and films [24]. Nevertheless, its performance can be further enhanced through the incorporation of nanofillers, such as silver nanoparticles (AgNPs), copper oxide (CuO), graphene oxide (GO), or zinc oxide (ZnO). These nanocomposites exhibit synergistic effects, improving both the mechanical durability and broad-spectrum antimicrobial properties of the coatings [25,26]. Shinde et al. have demonstrated strong antibacterial activity of CS-based coatings containing AgNPs against Staphylococcus aureus and *Escherichia coli* [27].

PCL, in contrast, is a semicrystalline aliphatic polyester characterized by good flexibility, thermal stability, and ease of processability, which make it a suitable candidate for the development of durable and adherent coatings [28]. When reinforced with nanofillers such as silver nanoclusters, bioactive glass particles, or essential-oil-loaded nanostructures, PCL-based coatings display enhanced antibacterial activity as well as long-term stability [29,30]. These hybrid systems offer the possibility of tailoring release kinetics and ensuring sustained antimicrobial protection while maintaining biocompatibility.

In this context, the present study focuses on the design, preparation, and in-depth characterization of chitosan- and poly(ε-caprolactone)-based antibacterial coatings incorporating chlorhexidine-loaded zirconium phosphate (ZrPCHX) nanoparticles. The coatings were applied using appropriate deposition techniques, including spray deposition with a double-action airbrush and brush coating for CS-based and PCL-based systems, respectively [31]. These approaches are in line with previous studies on airbrush-sprayed coatings and spray/atomized layer-by-layer films [32].

The study aimed to optimize both the formulation and deposition parameters to obtain uniform, adherent, and effective coatings with well-controlled thickness while systematically evaluating their morphological, physicochemical, and functional properties. A critical aspect of the investigation concerned the cytocompatibility of the developed nanocomposite coatings, assessed through in vitro assays, to confirm their safety for potential clinical use. Furthermore, their antibacterial efficacy was tested against a panel of clinically relevant pathogens, using standardized protocols (ISO 22196:2011) as well as newly custom-designed procedures simulating real hospital conditions. Overall, this integrated approach is intended to lay the groundwork for a sustainable and scalable strategy for the decontamination of hospital high-touch surfaces, providing continuous antimicrobial action at the point of contact and contributing to a reduction in healthcare-associated infections (HAIs).

## 2. Materials and Methods

### 2.1. Materials

Zirconium hydrogen phosphate (α-ZrP) was synthesized according to a conventional reflux method and used in its protonated form (acid capacity: AEC = 6.6 mmol g^−1^). Chlorhexidine digluconate (FW: 897.78 g/mol; 20 wt% aqueous solution) and propylamine (1 M aqueous solution) were of analytical grade and used as received. All experiments were carried out using deionized water with a conductivity of 1–30 μS.

Medium-molecular-weight chitosan powder (CS) was purchased from Sigma Aldrich. Its deacetylation degree, of approximately 77%, was measured using FTIR spectroscopy and the Sabnis and Block Methods [33]. Poly(ε-caprolactone) (PCL) pellets (CapaTM 6800, CAS 24980-41-4) were provided by Perstorp (Malmö, Sweden). Acetic acid (≥99.8%) and glutaraldehyde solution (GA; grade II, 25% in H_2_O) were provided by Sigma Aldrich (©2025 Merck KGaA, Darmstadt, Germany) as a crosslinking agent. Chloroform, Analar MORMAPUR reagent Ph. Eur., stabilized with approximately 0.6% of ethanol (CAS 67-66-3) was supplied by VWR Chemicals (Milan, Italy) as a solvent for preparing PCL-based suspensions. Distilled water was used as a solvent for active formulation. Polypropylene sheets (PP, Ref. 35463582; size: 50 × 100 cm; thickness: 1 mm) were purchased from Leroy Merlin (Naples, Italy). All reagents were used as received.

Murine dermal fibroblasts (L929) and human dermal fibroblasts (HDFs) (ATCC) were purchased from Merck (Milan, Italy). Dulbecco’s Modified Eagle’s Medium (DMEM), Foetal Bovine Serum (FBS), 1% non-essential amino acid (NEAA), antibiotic solution (streptomycin 100 µg/mL and penicillin 100 U/mL), and L-glutamine were purchased from Merck (Milan, Italy).

### 2.2. Synthesis of α-Zirconium Hydrogen Phosphate Intercalated with Chlorhexidine (ZrP-CHX)

The synthesis was carried out in three steps:

Step 1: Exfoliation and regeneration of α-ZrP.

A 10 wt% dispersion of α-ZrP in deionized water was prepared and subjected to vigorous mechanical stirring. Exfoliation was induced by adding a 1 M propylamine aqueous solution dropwise. The amount of propylamine corresponded to 50% of the exchangeable protons relative to the anion exchange capacity (AEC) of ZrP (3.3 mmol g^−1^). Upon completion of the addition, the dispersion was maintained under stirring for 1 h.

Subsequently, α-ZrP was regenerated in its acidic form by adding a single portion of 1 M HCl equal to four times the AEC. The resulting dispersion was ultracentrifuged at 10,000 rpm for 5 min, and the recovered paste was redispersed in deionized water to obtain a 10 wt% slurry. This washing–centrifugation cycle was repeated three times.

Step 2: Intercalation of chlorhexidine (CHX).

The washed ZrP was redispersed in deionized water to obtain a 10 wt% dispersion. A 20 wt% chlorhexidine digluconate solution was then added in an amount equimolar to the AEC of ZrP. The mixture was stirred mechanically for 4 h to allow intercalation of chlorhexidine molecules into the ZrP lamellae. The solid was isolated by Büchner filtration.

Step 3: Purification, drying, and conditioning.

The collected solid was washed by redispersing it in deionized water (10 wt%) and refiltering through a Büchner funnel. The washing cycle was repeated three times. The final washed product was dried in a ventilated oven at 80 °C until constant mass, then ground using a blade mill and sieved to 100 μm to yield the final hybrid material, hereafter referred to as ZrP-CHX.

### 2.3. Preparation of CS- and PCL-Based Active Films

Active films were prepared following a single general procedure, differing only in the solvent used for polymer dissolution.

CS-based active films were obtained by dissolving in distilled water containing about 2% of acetic acid, to achieve a pH = 4.5, keeping it under stirring and at room temperature. The (nano)composites were obtained by dispersing 5% wt/wt of active nanoparticles (ZrPCHX) directly in the aqueous solution of chitosan and subsequently by sonicating it for about 30 min. GA was added in a suitable amount (previously determined) to the aqueous dispersion of chitosan for the crosslinking of the polymeric film.

PLA-based active films were prepared by dissolving PCL pellets in chloroform under stirring at room temperature overnight. ZrPCHX nanoparticles were subsequently added at 5% by weight with respect to the polymer mass.

The CS- and PCL-based dispersions were then poured into plastic Petri dishes (diameter ≈ 5 cm) and dried under ambient temperature and humidity conditions until complete solvent evaporation, obtaining self-supporting films with a thickness of 25–35 μm, coded as follows: CS/ZrPCHX and PCL/ZrPCHX. The self-standing films were produced for the purpose of conducting characterization analyses. Subsequently, the corresponding formulations were employed to obtain coatings on substrates (with a thickness of approximately 10–15 μm). Pure CS and PCL films (without additives) were prepared and tested for comparative purposes.

### 2.4. Preparation of CS- and PCL-Based Active Coatings on PP Substrates

**Figure 1:**
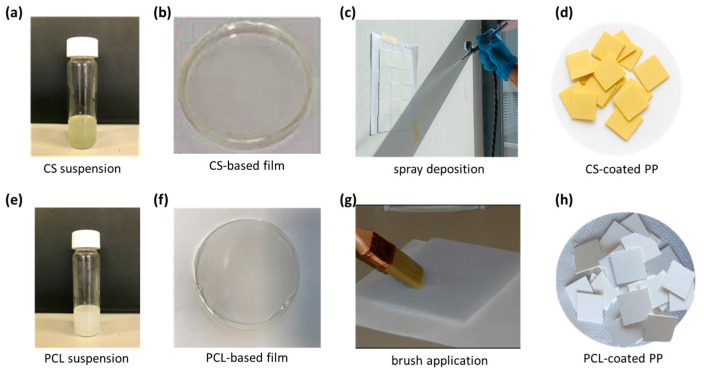
(**a**,**e**) CS- and PCL-based active formulation coating; (**b**,**f**) active self-standing films prepared for characterization purposes; (**c**,**g**) deposition technique for CS-based and PCL-based formulation; (**d**,**h**) CS-coated and PCL-coated PP substrates, respectively.

Both CS- and PCL-based formulations, loaded with ZrPCHX nanoparticles, were used to coat PP substrates (5 × 5 cm). In the case of CS-based nanomaterials, the suspensions were nebulized on the PP substrates by using a double-action airbrush, characterized by a unique control that allows the air flow and the amount of paint or colour released to be controlled independently. The PCL-based suspensions were directly deposited on the substrates using a specific brush for water-based paints (Figure 1). The obtained PP substrates coated with active CS- and PCL-based coatings were coded as follows: PP/CS/ZrPCHX and PP/PCL/ZrPCHX. For comparative purposes, PP substrates coated with CS and PCL not containing the active nanoparticles were prepared and coded as PP/CS and PP/PCL. All the obtained coatings had a nominal thickness of about 10–15 microns.

### 2.5. Chemical–Physical Characterization

#### 2.5.1. Synthesized ZrP-CHX

Morphological features were obtained by a LEO 1525 field-emission scanning electron microscope with an EDX Bruker probe.

Particle size distribution (PSD) was determined by a laser diffraction particle size analyser, Malvern Mastersizer 2000 (Alfatest, Milano, Italy). The samples were analysed in dry mode with Scirocco.

Thermogravimetric analysis (TGA) was performed in air with a ramp up of 10 °C/min by a Perkin Elmer STA 8000 simultaneous thermal analyser (Perkin Elmer, Milano, Italy).

FTIR spectra were recorded by using a Jasco 4600 FTIR spectrometer (Jasco Europe S.R.L., Lecco, Italy) in the range of 450–4000 cm^−1^. Each spectrum was a mean of 100 scans. The samples were analysed in tablet form, obtained by mixing KBr and 1% sample powder. An FTIR spectrum of pure KBr was always recorded and subtracted as background.

XRPD was performed with a Bruker D2 advance diffractometer (Bruker D2 PHASER 2nd generation (Bruker Italia srl, Milano, Italy)) operating at 30 kV and 10 mA, a step size of 0.020 2 q degrees/step, and a time of 1.00 s per step using copper K-α (Cu K-α) radiation and a multistrip LYNXEYE SSD160 detector. DIFFRACTPLUS EVA V4.2.2 software was used to perform the manipulations.

#### 2.5.2. Films and Coatings

WAXS analysis was performed to evaluate the structural modifications of the ZrPCHX nanoparticles following the delamination process and their incorporation into the chitosan-based matrix. The diffractograms were collected using an Anton Paar SAXS camera equipped with a 2D imaging plate detector. Cu Kα radiation (λ = 1.5418 Å) was generated by a Philips PW3830 sealed-tube source operating at 40 kV and 50 mA, with slit collimation. The samples were scanned for 10 min. The degree of structural ordering and the possible intercalation/exfoliation of the lamellar filler within the polymeric matrix were investigated by tracking changes in the basal reflections.

The morphological analysis of pristine and nanocomposite CS- and PCL-based materials was performed by using an FEI Quanta 200 FEG scanning electron microscope (ESEM) (FEI, Eindhoven, The Netherlands) in high-vacuum mode by using an accelerating voltage within a 10–20 kV range and a secondary electron detector (Everhart–Thornley detector). Before the analysis, dried specimens were mounted on aluminium stubs by means of carbon adhesive disks and coated with a thin layer (about 10 nm thick) of a Au-Pd alloy by means of an Emitech K575 sputter coating system (Quorum Technologies Ltd., Ashford, UK).

Contact angle measurements were carried out by using a DataPhysics OCA 20 apparatus. Distilled water was dropped onto at least 10 different sites on each sample, and the static contact angle was reported as the average value from the measured ones.

Fourier-transform infrared spectroscopy (FT-IR) spectra were recorded at room temperature by using an FT-IR spectrometer (model Frontier Dual Ranger, PerkinElmer, Shelton, CT, USA) in attenuated total reflectance (ATR) mode from 650 to 4000 cm^−1^. Spectra were recorded at 4 cm^−1^ resolutions, and the reported results are the average of 64 scans.

Adhesion was performed by the Type Test, according to ASTM D3359—Method A—by applying and removing pressure-sensitive transparent tape (number “# 600” of 3M with a width of 10″) over cuts made in the film on different substrates. In detail, on a 10 × 10 cm^2^ area, free of surface imperfections, different cuts (each about 10 mm) were made through the film up to the support, creating a lattice at right angles on the surface.

### 2.6. Biocompatibility Evaluation

#### 2.6.1. Cell Cultures

Cytotoxicity studies on CS- and PCL-based materials were carried out using murine L929 and human dermal fibroblasts. L929 fibroblasts and HDFs were cultured in 75 cm^2^ cell culture flasks in Dulbecco’s Modified Eagle’s Medium (DMEM) supplemented with 20% FBS, 1% NEAA, antibiotic solution, and 2 mM L-glutamine. The cells were maintained in culture at 37 °C, 5% CO_2_, and 95% humidity. Before biological testing, the samples were sterilized under UV rays for 2 h with ethanol (70 *v*/*v*%).

#### 2.6.2. Cytotoxicity Tests

The cytotoxicity of CS and PCL as 2D substrates was assessed using indirect tests, as detailed below.

Indirect test: The assessment followed ISO 10993-5, which provides test procedures for evaluating the in vitro cytotoxicity of medical devices. It was employed to test biocompatibility. To determine biocompatibility, an elution test was performed by incubating the materials in sterile DMEM (extraction vehicle) at a ratio of 0.1 g/mL at 37 °C, in accordance with ISO 10993-12 guidelines. After 24 h of incubation (elution time), the conditioned media (eluants) were collected, and 200 µL was transferred into 96-well plates previously seeded with HDFs and L929 at 80% confluence. The plates were incubated for an additional 24 h (exposure time) [34]. After incubation at 37 °C and 5% CO_2_, the cells were observed under an optical microscope (10× magnification) to identify signs of cytotoxicity over time (e.g., changes in cell morphology, size, or lysis). Cell proliferation and viability were subsequently assessed using the Alamar Blue assay.

#### 2.6.3. Statistical Analysis

All quantitative experiments were performed in triplicate, and the results are expressed as the mean ± standard deviation (SD). Statistical analysis was undertaken using GraphPad Prism^®^, version 5.00 (GraphPad Software, La Jolla, CA, USA, www.graphpad.com). Data were analysed using a Student’s *t*-test, a one-way ANOVA, and a Bonferroni post-test (parametric methods). Groups differences of *p* * < 0.01, *p* < 0.001, and *p* # < 0.0001 were considered statistically significant.

### 2.7. Antibacterial Activity Evaluation

The antibacterial activity of the test samples was evaluated through in vitro assays performed in compliance with ISO 22196:2011 [35].

This technical standard defines a quantitative method for determining the antibacterial activity of non-porous surfaces, including polymeric materials treated with antibacterial agents. For in vitro testing, a Gram-negative bacterium (*Escherichia coli*, ATCC^®^ 8739) and a Gram-positive bacterium (Staphylococcus aureus, ATCC^®^ 6538P) were employed.

In accordance with the guidelines provided by the ISO 22196 standard, the Gram-positive bacterium (*Staphylococcus aureus*, ATCC^®^ 6538P) and the Gram-negative bacterium (*Escherichia coli*, ATCC^®^ 8739) were cultured on Nutrient Agar (Oxoid Ltd., Basingstoke, UK) and incubated at 35 ± 1 °C for 18–20 h. In addition to the strains specified by ISO 22196, in vitro assays were also performed using the following clinically relevant bacterial strains: methicillin-resistant Staphylococcus aureus (MRSA, ATCC^®^ 43300), *Klebsiella pneumoniae* (ATCC^®^ BAA-1705), and *Acinetobacter baumannii* (ATCC^®^ 19606).

The bacterial strains, retrieved from frozen stock cultures, were initially inoculated into screw-capped tubes containing Nutrient Agar and incubated at 35 ± 1 °C for 18–20 h. Subsequently, a loopful of each culture was transferred into tubes containing Nutrient Broth (NB) diluted to 1:500 in deionized water and incubated overnight. Prior to inoculation, the bacterial suspensions were refreshed in NB (1:500 dilution) and incubated under orbital shaking at 35 ± 1 °C until the exponential growth phase was reached. The cultures were then centrifuged (2500 rpm, 10 min), and the resulting pellets were resuspended in phosphate-buffered saline (PBS). The bacterial concentration was adjusted spectrophotometrically to reach a target concentration of approximately 2.5 × 10^5^ and 10 × 10^5^ colony-forming units (CFUs)/mL, which was used as the test inoculum.

Prior to testing, all specimens (standard dimensions: 50 mm × 50 mm × 2 mm) were decontaminated using 95% ethanol and subsequently air-dried in a biological safety cabinet for 30 min. A volume of 400 µL of bacterial inoculum was deposited onto both the PP/CS/ZrPCHX and PP/PCL/ZrPCHX specimens and the polypropylene (PP) specimens, the latter serving as controls.

Antibacterial activity was assessed at time zero (T0), immediately after inoculation, and after 1 (T1), 2 (T2), and 24 h (T24) of incubation. After incubation, each specimen was transferred into a conical tube containing 10 mL of neutralizing solution (D/E Neutralizing Broth, BD Difco™) and vortexed for few minutes to detach the bacteria. Serial dilutions of the resulting suspension were plated on Plate Count Agar (PCA, Oxoid Ltd.) and incubated at 35 ± 1 °C for 24–48 h. Bacterial colonies were then enumerated, and the number of colony-forming units (CFUs) per cm^2^ was calculated and expressed as decimal logarithms.

## 3. Results

### 3.1. Structural Characterization of ZrPCHX Powders

**Figure 2:**
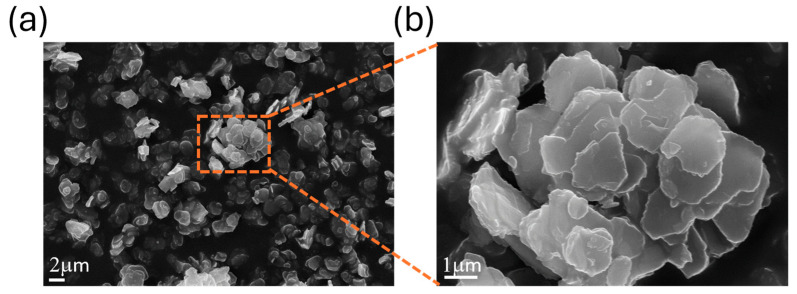
SEM micrographs of ZrP-CHX at (**a**) 5000× and (**b**) 25,000× magnification.

The SEM micrographs of the ZrP–CHX hybrid (Figure 2a,b) reveal that the material retains the typical lamellar morphology of zirconium hydrogen phosphate. The particles exhibit pseudo-hexagonal platelets with micrometric lateral dimensions, consistent with the crystalline habit of α-ZrP. At lower magnification, the powder appears as an assembly of agglomerated lamellar crystallites, whereas higher-magnification images highlight the stacked platelet architecture, with well-defined edges and smooth basal surfaces.

The preservation of this layered morphology after chlorhexidine intercalation confirms that the treatment does not compromise the structural integrity of the ZrP lamellae. Instead, the platelets remain distinct and well structured, suggesting that intercalation occurs predominantly within the interlayer galleries rather than through exfoliation or extensive disruption of the crystal habit. This morphological stability is consistent with the XRPD results, which indicate retention of long-range lamellar order, and supports the suitability of ZrP-CHX as a structurally robust hybrid filler for polymer composites.

**Figure 3:**
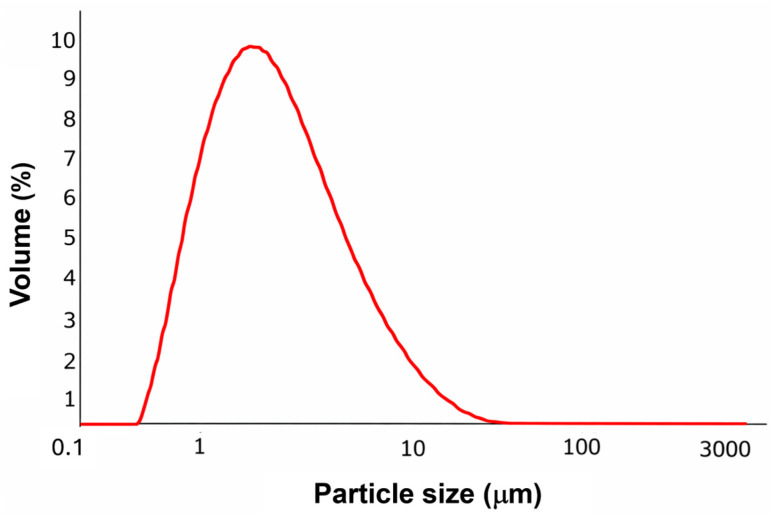
Particle size distribution of ZrPCHX.

The PSD of ZrP-CHX (Figure 3) exhibits a narrow, unimodal profile, indicating good homogeneity of the powder after milling and sieving. The distribution is centred around micrometric particle sizes, with characteristic values of d(0.1) = 1.06 μm, d(0.5) = 2.12 μm, and d(0.9) = 4.85 μm. This indicates that 50% of the particle volume lies below approximately 2 μm and that most of the sample (90%) consists of particles smaller than 5 μm.

Such a narrow PSD is consistent with the lamellar morphology observed by SEM, where plate-like crystallites fragment into micrometric entities while preserving their structural identity. The reduced presence of large aggregates and the absence of a coarse particle tail suggest effective dispersion and size reduction during post-synthesis processing.

This controlled micrometric particle size is particularly advantageous for applications where uniform dispersion within polymer matrices is required—for example, in extrusion-grade polycaprolactone composites—ensuring homogeneous filler distribution and reproducible mechanical and functional performance.

**Figure 4:**
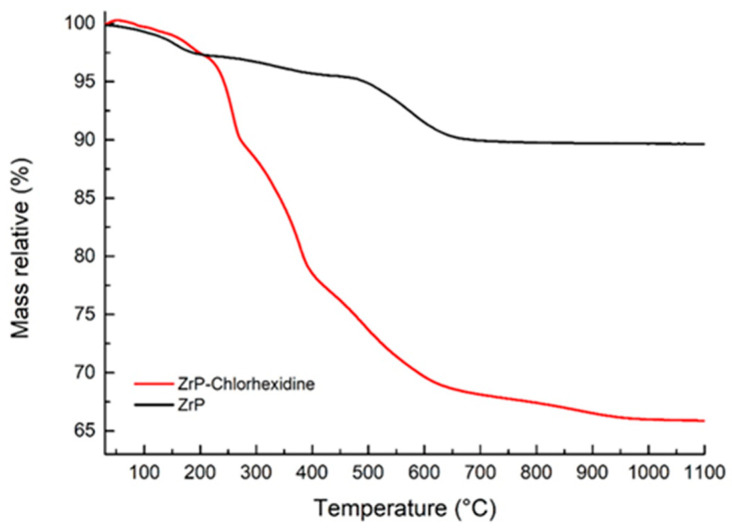
Thermogravimetric curves of pristine zirconium hydrogen phosphate (ZrP, black) and the ZrP–chlorhexidine intercalation compound (ZrP-CHX, red).

The TGA curve of pristine ZrP (black line) shows two main weight-loss events (Figure 4). The first, occurring between approximately 20 and 200 °C, is ascribed to the release of physiosorbed and interlayer water. A second, broader mass-loss step between about 200 and 800 °C is associated with the condensation of HPO_4_ groups, ultimately leading to the formation of zirconium pyrophosphate. Above 800 °C, the residual mass remains nearly constant at ca. 90 wt%, in agreement with the high thermal stability of the inorganic framework.

The ZrP–CHR sample (red line) exhibits an initial weight loss in the 20–200 °C region due to the removal of water, followed by two major steps between 200 and 800 °C. These latter events are attributed to the thermal degradation of chlorhexidine together with the progressive condensation of phosphate groups to zirconium pyrophosphate. At high temperatures, the final residue is about 65–66 wt%, substantially lower than that of pristine ZrP as a consequence of the combustion of the organic fraction.

By comparing the total mass loss of ZrP–CHR (≈34 wt%) with that of pristine ZrP (≈10 wt%), the organic contribution can be estimated as ~24 wt%. This value is consistent with a chlorhexidine loading of 23.7 wt% in the intercalation compound, confirming that the hybrid material contains a significant amount of drug while preserving an inorganic backbone stable up to high temperatures.

A comparison of the TGA profiles of pristine ZrP and the ZrP-CHX hybrid clearly shows that intercalation significantly enhances the thermal stability of chlorhexidine. Whereas free chlorhexidine typically undergoes rapid thermal degradation at relatively low temperatures, its incorporation into the ZrP interlayer galleries shifts its decomposition to markedly higher temperature intervals. This stabilizing effect is commonly attributed to the strong confinement within the lamellar structure, electrostatic interactions with acidic phosphate groups, and restricted molecular mobility, all of which hinder volatilization and oxidative breakdown.

Importantly, the inorganic ZrP framework remains highly stable throughout the entire thermal range, and the hybrid material preserves a substantial inorganic residue even above 800 °C. The combination of an intact inorganic scaffold and a thermally protected organic phase make ZrP-CHX particularly suitable as a functional filler in thermally processed polymer matrices, including melt-processable systems such as polycaprolactone (PCL). The elevated decomposition onset of the intercalated chlorhexidine ensures that the hybrid filler can withstand typical extrusion temperatures without premature drug degradation, thereby enabling the development of antimicrobial, extrusion-grade polymer composites with controlled release properties.

**Figure 5:**
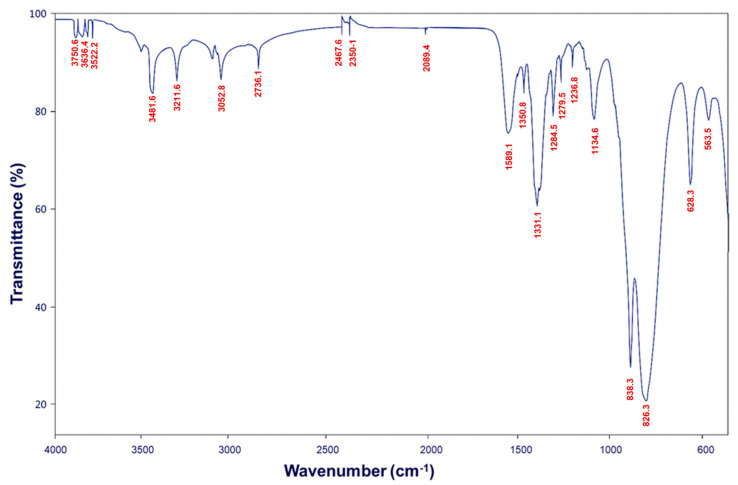
FT-IR spectrum of ZrP–CHX hybrid.

The FT-IR spectrum of the ZrP–CHX hybrid (Figure 5) exhibits the characteristic vibrational peaks of both zirconium hydrogen phosphate and chlorhexidine, confirming the coexistence of the inorganic lamellar framework and the intercalated organic molecule.

The main absorption bands associated with ZrP are well preserved, including (i) a broad band in the 3000–2500 cm^−1^ region attributed to P–OH stretching vibrations; (ii) intense and complex bands in the 1100–900 cm^−1^ region, corresponding to asymmetric and symmetric P–O stretching modes; and (iii) peaks around 730–700 cm^−1^, typically assigned to P–O–Zr vibrations and lattice deformation modes.

Superimposed on these signals, several bands characteristic of chlorhexidine are also observed, indicating successful intercalation within the ZrP matrix. Notable features include N–H stretching vibrations at approximately 3300–3200 cm^−1^, C–H stretching bands of aromatic and aliphatic groups in the 2950–2850 cm^−1^ region, the C=N stretching vibration of the biguanide moiety near 1630–1610 cm^−1^, aromatic ring vibrations in the 1500–1450 cm^−1^ range, and additional N–H and C–N modes between 1350 and 1200 cm^−1^.

The simultaneous presence of phosphate-related vibrations and diagnostic chlorhexidine bands confirms the formation of a true hybrid material, in which CHX is retained within or on the ZrP layers without disruption of the inorganic lattice. Minor shifts and broadening of the phosphate bands suggest the occurrence of hydrogen bonding and electrostatic interactions between the biguanide groups of CHX and the acidic P–OH groups of ZrP.

**Figure 6:**
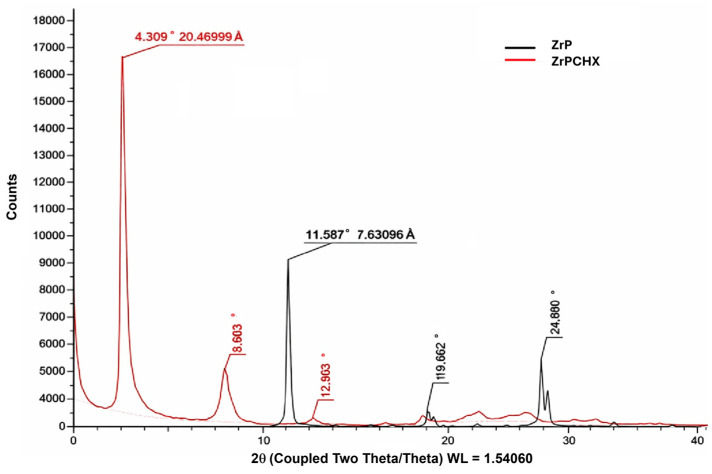
XRPD for ZrP (black line) and ZrP-CHX (red line), while the numerical values indicate the corresponding d-spacing (Å).

The XRPD patterns of pristine ZrP and ZrP-CHX (Figure 6) exhibit clear differences, providing direct evidence of chlorhexidine incorporation within the layered zirconium hydrogen phosphate structure.

For the starting α-ZrP, the diffraction pattern shows a very intense basal (001) reflection at 2θ ≈ 11.6°, corresponding to an interlayer distance d_001_ ≈ 7.63 Å, fully consistent with values reported for crystalline zirconium hydrogen phosphate. Additional reflections at 2θ ≈ 19.9° and 24.8° (assigned to higher-order and hk planes) confirm well-ordered lamellar stacking and good in-plane crystallinity, with no detectable secondary phases.

In the diffraction pattern of ZrP-CHX, the original basal peak at 11.6° disappears and is replaced by a new, intense reflection at 2θ ≈ 4.3°, corresponding to d_001_ ≈ 20.5 Å. This represents an expansion of approximately 13 Å relative to pristine ZrP, clearly indicating the insertion of bulky chlorhexidine molecules between the ZrP layers. The presence of additional reflections at 2θ ≈ 9.0°, 12.9°, 17.1°, 19.7–22.0°, and 24.2–27.0° indicates that the material remains lamellar, although with a higher degree of structural disorder, as suggested by peak broadening and the reduced relative intensities with respect to the inorganic precursor.

Overall, the marked shift of the (001) peak from 11.6° to 4.3°, together with the disappearance of the original basal reflection and the appearance of a new set of correlated reflections, provides strong evidence for the successful intercalation of chlorhexidine within the interlayer galleries of ZrP. The resulting hybrid material (ZrP-CHX) exhibits long-range lamellar order, with only a marginal presence of non-intercalated ZrP, if any.

### 3.2. Morphological and Physicochemical Characterization of CS- and PCL-Based Films

The ZrPCHX active filler and the ZrPCHX-based CS film were compared by WAXS analysis to evaluate the structural and morphological properties of the composite film.

**Figure 7:**
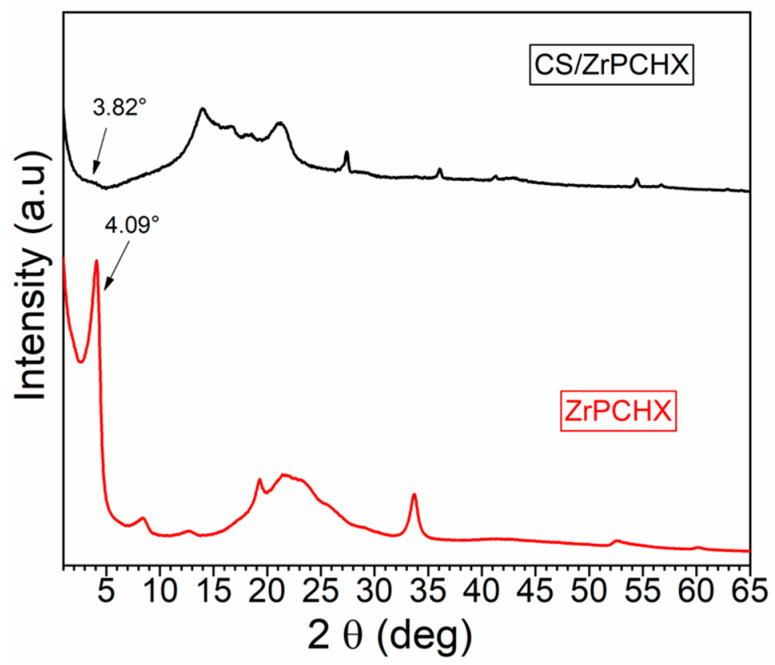
WAXS results of ZrPCHX before and after exfoliation.

In Figure 7, WAXS analysis reveals the that the zirconium phosphate retained its original lamellar organization with chlorhexidine molecules intercalated between the layers, as previously discussed. By contrast, the ZrPCHX composite shows a marked reduction in peak intensity and significant broadening of the reflections, accompanied by a shift of the main peak to a lower angle (2θ = 3.82°, d = 23.1 Å). These changes suggest partial exfoliation of the layered structure, which is promoted by ion exchange processes likely occurring during ZrPCHX powder dispersion in the polymeric aqueous formulation. This led to the partial deintercalation of chlorhexidine and disruption of long-range interlayer order [36].

Furthermore, the broadening of the basal reflection and the disappearance of the high-angle peaks suggest a loss of three-dimensional periodicity, indicating the formation of nanoscale domains. These findings confirm that simple aqueous treatment effectively delaminates ZrPCHX, producing a nanostructured form that is more compatible with polymer matrices and potentially better suited to tailor its final functionality.

The observed increase in the interlayer distance is consistent with successful intercalation of the active molecule into the ZrP galleries, a mechanism already reported for other therapeutic agents such as doxorubicin, intercalated via direct ion exchange into ZrP nanoplatelets [37].

The delamination of ZrP–CHX nanoparticles increases the exposure of intercalated C<HX molecules at the coating surface, enhancing the availability of active sites for bacterial contact. This structural effect, combined with polymer–membrane interactions, could contribute to the rapid and effective antimicrobial performance observed in the developed systems.

**Figure 8:**
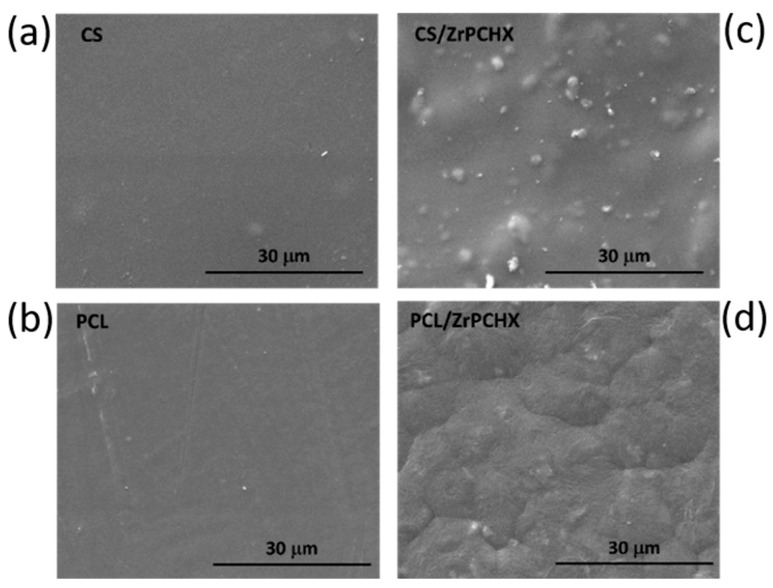
SEM images of pristine (**a**) CS and (**b**) PCL and their corresponding (**c**) CS/ZrPCHX and (**d**) PCL/ZrPCHX composites.

To assess the effective incorporation of ZrPCHX nanoparticles, CS- and PCL-based films were first investigated in terms of their morphology and microstructure by SEM analysis (Figure 8). This characterization provides direct evidence of the active filler (ZrPCHX) dispersion within the polymeric matrices (CS and PCL) and allows assessment of its influence on the final surface features of the coatings. Figure 3 shows SEM images of neat CS and PCL films and their corresponding nanocomposites containing ZrPCHX.

The pristine CS film exhibits a smooth, homogeneous, and compact surface, typical of well-formed chitosan-based coatings. Upon incorporation of ZrPCHX, the CS/ZrPCHX film shows an uneven distribution of active particles, appearing as irregularly shaped agglomerates scattered over the surface and attributable to the embedded nanoparticles. Importantly, no evident phase separation is observed, indicating good compatibility between the CS matrix and the inorganic–organic hybrid filler. Despite the presence of agglomerates, the overall particle distribution suggests effective polymer–filler interactions, likely driven by hydrogen bonding and electrostatic interactions between the protonated amino groups of chitosan and the phosphate groups of ZrPCHX.

In the case of PCL-based films, the neat PCL sample displays a smooth surface with faint processing-related features, consistent with the semicrystalline nature of the polymer. After incorporation of ZrPCHX, a marked change in surface morphology is observed: the PCL/ZrPCHX film exhibits a uniformly roughened and textured surface, with the nanoparticles appearing homogeneously distributed within the polymer matrix. Unlike the CS system, the particles are less distinctly protruding from the surface, suggesting a higher degree of embedding within the PCL phase, possibly due to differences in polymer viscosity and solidification behaviour during film formation.

### 3.3. Wettability Analysis

**Figure 9:**
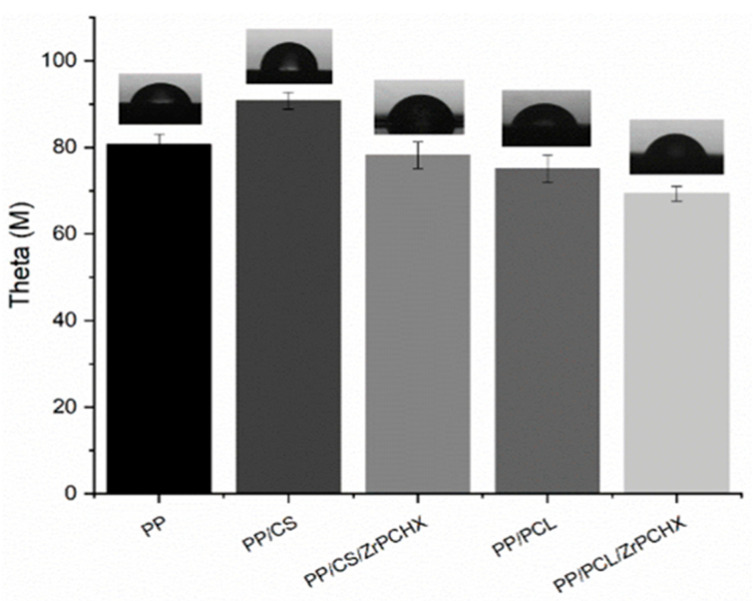
Contact angle for PP substrate and PP substrate coated by CS-, PCL-, CS/ZrPCHX-, and PCL/ZrPCHX-based coatings.

Contact angle measurements were carried out to study the effect of ZrPCHX on film wettability. The results obtained for CS- and PCL-based coatings are shown in Figure 9, along with the corresponding error ranges. For each sample, the figures also show the most significant images acquired during the different tests. The presence of the CS-based coating on the PP surface leads to an increase in its hydrophobicity (e.g., 90.8° for PP/CS and 80.7° for PP). Otherwise, the addition of ZrPCHX nanoparticles makes the polymeric films more hydrophilic, as can be seen from the lower contact angle values and the way the water drop adheres to the surface of the films (78.2° for CS/PP/ZrPCHX). The PCL-based coatings induce a higher wettability towards the substrate; in fact, the contact angle values are 75.0° and 69.3° for PP/PCL and PP/PCL/ZrPCHX, respectively.

**Figure 10:**
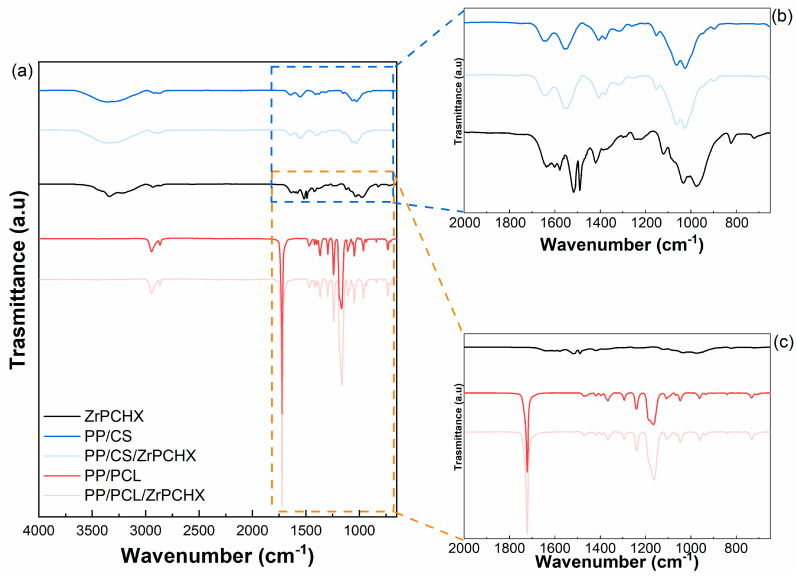
(**a**) FTIR spectra of pristine ZrPCHX nanoparticles (black line) and PP/CS (blue line), PP/CS/ZrPCHX (light blue line), PP/PCL (red line), and PP/CS/ZrPCHX (light red line) films and (**b**,**c**) FT-IR spectra magnification from 1880 to 650 cm^−1^.

FTIR analyses were performed to investigate the potential interfacial interaction between ZrPCHX nanoparticles and both polymeric matrices. For this purpose, FTIR experiments of ZrPCHX-loaded CS- and PCL-based coatings were performed and compared to the unloaded CS and PCL and ZrPCHX powder. All spectra are shown in Figure 10a–c.

The ZrPCHX spectrum (black curve) shows characteristic NH stretching amine in the region between 3500 and 3250 cm^−1^ [38], the N-H stretching of secondary amine salts at 2542 and 2956 cm^−1^ [39], and the N-H bending of secondary aromatic amine at 1519.80 cm^−1^ [40]. In 1690–1640 cm^−1^ frequency range, the C=N stretching vibrational bands are related to both the aromatic guanidine (ArNHC(=N-H)NHAr) and (CH_3_)_2_NC(=N-H)C(CH_3_)_2_ [41]. The bands at 1670–1626 cm^−1^ are due to C=C stretching vibrations, and C-C alkaline stretching appears in the range of 1400–600 cm^−1^ [42]. C-N secondary aromatic amine stretching is in the 1300–1000 cm^−1^ frequency range [43]. The presence of ZrPCHX does not affect the chemical behaviour of CS and PCL backbones; in fact, the spectra related to both CS- and PCL-based coatings show all the characteristic peaks related to the polymer matrices [44,45].

### 3.4. Coating Adhesion Tests

**Figure 11:**
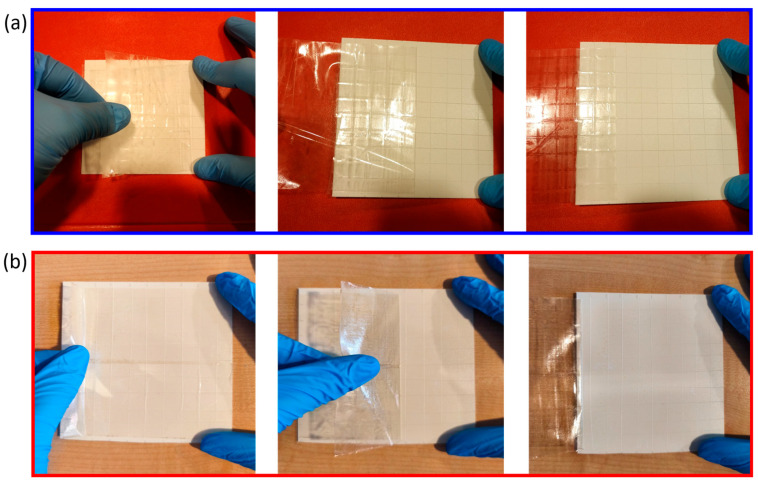
(**a**) CS/ZrPCHX and (**b**) PCL/ZrPCHX coating films after cross-cut adhesion testing according to ASTM D3359, showing intact lattice patterns without evidence of peeling or flaking, corresponding to the highest adhesion rating (5B).

The adhesion properties of the developed coatings were evaluated using a standardized cross-cut test, commonly employed to determine the resistance of paints, varnishes, and polymeric films to detachment or peeling from the underlying substrate (Figure 11). In this method, a lattice pattern of cuts was made through the coating layer down to the substrate, followed by the application and rapid removal of a pressure-sensitive adhesive tape. The extent of coating removal from the cut areas was then examined visually to assess film adhesion.

The obtained results clearly indicated that both CS-based (CS/ZrPCHX) and PCL-based (PCL/ZrPCHX) coatings did not show any detachment, flaking, or visible defects around the lattice cuts, confirming the strong interfacial bonding between the coating and the substrate. In fact, according to ASTM D3359—Method B—which provides a standardized classification ranging from 0B (poor adhesion, >65% removal) to 5B (excellent adhesion, no removal), both coating formulations achieved the highest rating (5B). This outcome demonstrates the excellent adhesion of the coatings to the polypropylene substrates due to the robustness of the coating–substrate interaction, thus confirming their suitability for practical applications, where long-term adhesion is a critical requirement.

### 3.5. Coating Resistance to Washing/Disinfection and Removability

Since these coatings have been specifically designed to coat and preserve high touch surfaces in hospital environments, it is crucial to achieve a balance between durability and sustained bactericidal activity but also controlled removability. This removability is essential not only to allow replacement of the coating with a more effective or updated version when needed but also to avoid causing permanent damage to the underlying hospital furniture and equipment.

**Figure 12:**
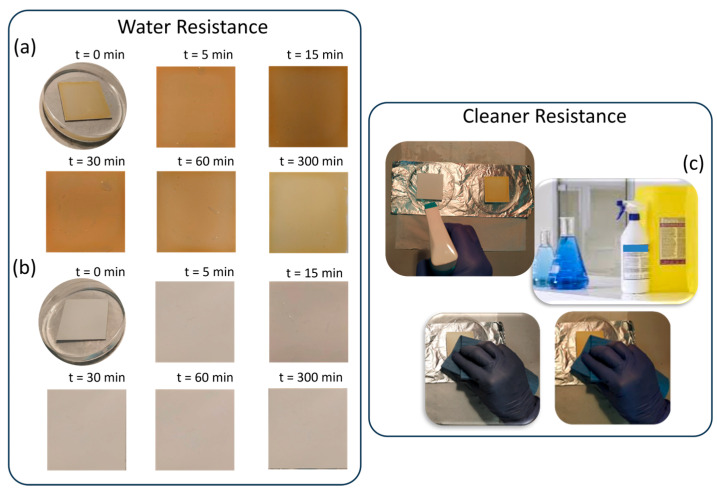
Water resistance at chosen time intervals of (**a**) CS-based and (**b**) PCL-based coatings. (**c**) Resistance against common hospital cleaners.

To this aim, the durability of the developed coatings under conditions relevant to practical applications was thoroughly evaluated, with particular focus on resistance to water, routine cleaning, and mechanical stress (Figure 12). For the water resistance test, coated substrates were immersed in Petri dishes containing liquid water and examined at predetermined time intervals. After five hours of continuous immersion, no visible detachment, blistering, or swelling was observed, confirming that both CS- and PCL-based coatings exhibited excellent stability in aqueous environments and still maintained strong adhesion to the substrate.

In addition to water stability, the resistance of the coatings to routine cleaning procedures was evaluated using commonly employed hospital disinfectant and cleaning sponges. Following repeated cleaning cycles, both types of coatings (CS/ZrPCHX and PCL/ZrPCHX) remained firmly attached to the substrate, indicating their suitability for environments where frequent disinfection is required.

**Figure 13:**
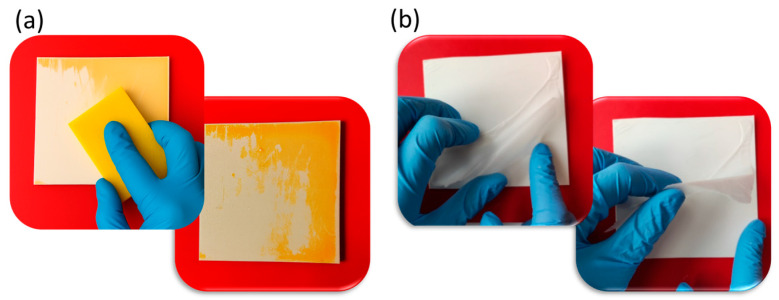
(**a**) Coating removal: progressive erosion by mechanical abrasion of chitosan film (**b**) vs. PCL film completely peeling off at localized “non-contact point”.

Moreover, coating removability was evaluated by subjecting the obtained CS- and PCL-based films to mechanical abrasion or localized stress, revealing clear differences between the two systems (Figure 13). While chitosan-based coatings could be gradually removed through mechanical abrasion with an abrasive sponge in the presence of water, PCL-based coatings displayed a tendency to peel off completely once detachment was initiated at a non-contact point. These observations highlight the importance of polymer matrix selection in determining the mechanical robustness of the coatings, with chitosan films providing partial resistance to abrasion and PCL films being more susceptible to peeling under localized stress.

### 3.6. Biocompatibility Studies

**Figure 14:**
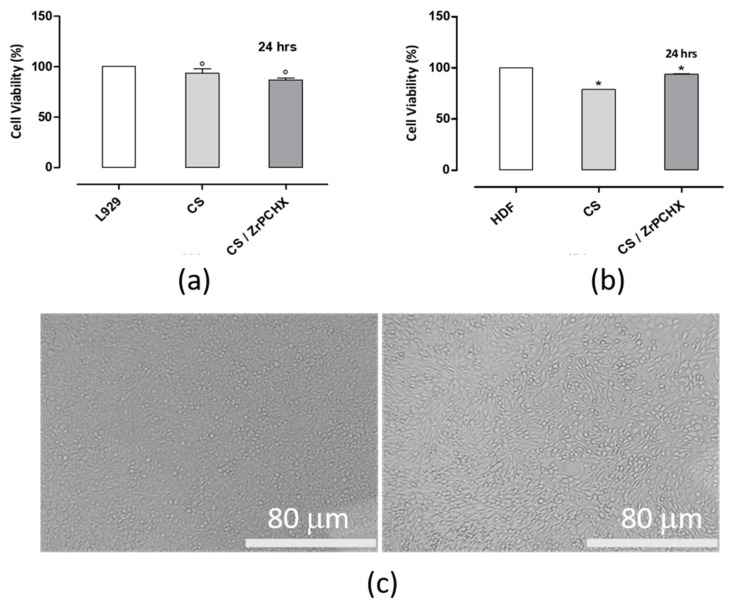
Indirect cytotoxicity test. Alamar Blue assay evaluating (**a**) L929 and (**b**) HDF cell viability after exposure to CS-conditioned media following 24 h of elution and 24 h of in vitro incubation. Optical micrographs (**c**) show L929 cell morphology after 24 h of treatment with CS-conditioned media compared to the L929 control.; ° *p* < 0.001 and * *p* < 0.01 vs. L929 and HDF control plate, respectively. Scale bar = 80 µm.

Human dermal fibroblasts (HDFs) and murine fibroblasts (L929) were used as physiologically relevant in vitro models to evaluate the cytotoxicity of the 2D substrates studied. Biocompatibility was evaluated using an indirect cytotoxicity assay with L929 cells, which demonstrated that the CS 2D substrates did not release any cytotoxic components into the conditioned culture media, as no harmful effects on L929 cell proliferation were observed after 24 h of culture. As shown in Figure 14, the materials exhibited good biocompatibility after 24 h of incubation (Figure 14a), further confirmed by microscopic observations that showed normal L929 cell morphology after exposure to CS-conditioned culture media (Figure 14c).

For HDFs (Figure 14b), a slight reduction in cell viability was observed in response to CS-conditioned culture media; however, this decrease remained within the acceptable threshold defined by ISO 10993-5, indicating that the materials can still be considered non-cytotoxic. This slight reduction may be attributed to the greater sensitivity of primary human cells compared to immortalized murine fibroblasts, or to transient interactions between residual ions or surface groups released from CS substrates. Overall, these results confirm the good cytocompatibility of 2D CS materials and support their potential suitability for biomedical applications involving direct or indirect contact with fibroblast cells.

**Figure 15:**
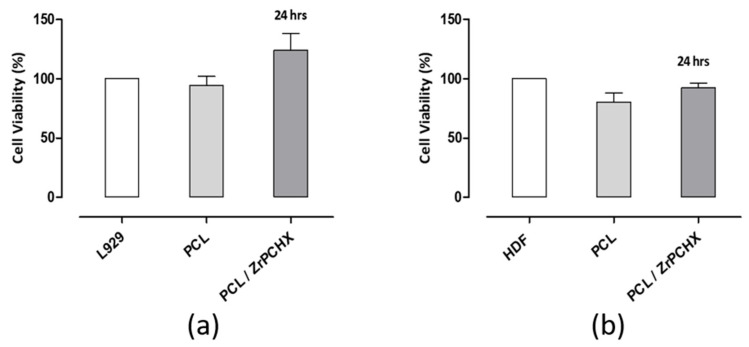
Indirect cytotoxicity test. Alamar Blue assay evaluating (**a**) L929 and (**b**) HDF cell viability after exposure to conditioned media from PCL and PCL/Zr-PCHX substrates following 24 h of elution and 24 h of in vitro incubation.

Furthermore, the cytotoxicity of pure PCL and PCL loaded with Zr-PCHX nanoparticles was evaluated using L929 and HDF cells. The results showed that, compared to CS substrates, both pure PCL and PCL/Zr-PCHX composites exhibited good biocompatibility for both cell types (Figure 15a,b). In particular, while CS substrates showed a slight reduction in HDF cell viability, although within the ISO 10993-5 threshold, PCL-based materials maintained constantly high cell viability for both L929 and HDF cells. This suggests that the PCL matrix, whether or not loaded with Zr-PCHX nanoparticles, provides a more inert environment for fibroblasts, potentially due to lower release of reactive species or reduced interactions with cell membranes compared to CS substrates. These results highlight the good cytocompatibility of PCL-based materials and reinforce their potential suitability for biomedical applications involving both direct and indirect contact with fibroblasts.

### 3.7. Antibacterial Activity Studies

The antibacterial activity of the PP/CS/ZrPCHX and PP/PCL/ZrPCHX samples was evaluated in accordance with the calculation method outlined by the ISO 22196 standard. The reduction value (R) in bacterial colony numbers was determined by quantifying the mean decrease in the number of viable bacterial cells on the tested specimens compared to the control (polypropylene, PP), as calculated using the following equation:

R = (Ut − U_0_) − (At − U_0_) = Ut − At (1)

where

U_0_ represents the average of the common logarithm of the number of viable bacteria (CFUs/cm^2^) recovered from the PP specimens immediately after inoculation (T_0_);
Ut indicates the average of the common logarithm of the number of viable bacteria (CFUs/cm^2^) recovered from the PP specimens after 24 h of incubation;
At is the average of the common logarithm of the number of viable bacteria (CFUs/cm^2^) recovered from the antibacterial coating specimens after 24 h of incubation.

Log-transformed data were employed for the analysis of bacterial counts. Antibacterial activity was attributed to specimens that achieved a reduction value (R) of ≥2 logarithmic units per cm^2^ of surface (Log_10_ CFUs/cm^2^) within a 24 h contact period.

**Figure 16:**
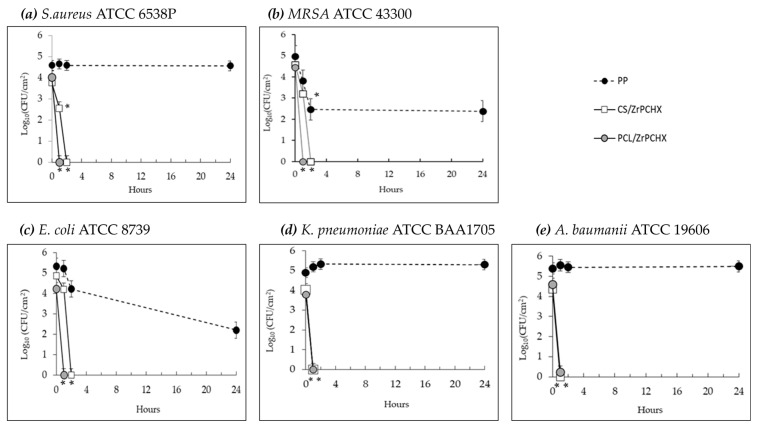
Time-dependent survival of two Gram-positive bacterial strains, (**a**) *S. aureus* and (**b**) MRSA, and three Gram-negative bacterial strains, (**c**) *E. coli*, (**d**) *K. pneumoniae*, and (**e**) *A. baumanii*, on PP (- -•- -) and specimens coated with CS/ZrPCHX (─□─) or PCL/ZrPCHX (─
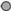
─) antibacterial films. Each point indicates the mean number of viable colonies per group of isolates. The number of bacteria was quantified immediately after inoculation and after 1, 2, and 24 h of incubation compared to the control (PP). Mean value of at least 3 experiments ± SD is presented. Differences with respect to controls were defined as statistically significant at *p* * < 0.01.

The in vitro test results, obtained according to the experimental procedure described in the ISO 22196:2011 standard, are shown in Figure 16.

Both antibacterial coatings (CS/ZrPCHX and PCL/ZrPCHX) exhibited antibacterial activity against all tested bacterial strains, although with different exposure times providing an insight into the rapidity of the coating antimicrobial response. The antibacterial coatings based on polycaprolactone (PCL) showed a more rapid onset of action compared to those based on chitosan (CS). In all in vitro assays conducted on PCL/ZrPCHX specimens, a consistent bactericidal activity (≥3 log10 reduction in CFUs) was observed within one hour of contact (t1) with the bacterial inoculum, across all tested strains. In the case of the chitosan-based specimens (CS/ZrPCHX), significant antibacterial activity was also observed, with a rapid and marked bactericidal effect observable as early as one hour after exposure to the antibacterial coating. This rapid bactericidal effect was, however, limited to the Gram-negative strains Klebsiella pneumoniae and Acinetobacter baumannii. Regarding *Escherichia coli* and the two Gram-positive strains (*Staphylococcus aureus* and MRSA), the chitosan-based coating exhibited bactericidal efficacy following two hours of exposure (t2) to the bacterial inoculum.

Table 1 reports the mean reduction values (R) in colony-forming units (CFUs) of the bacterial strains tested after contact with the PCL/ZrPCHX and CS/ZrPCHX specimens measured at different exposure times (t1, t2, and t24). The results obtained from tests performed on PCL-based specimens demonstrated substantial bactericidal efficacy following one hour of exposure to the bacterial inoculum, consistently across all five tested strains. Specifically, a mean reduction value (R) of 4.48 log_10_CFUs/cm^2^ was recorded, with minimum and maximum values of R = 3.83 and 5.0 log_10_ CFUs/cm^2^, respectively. Regarding the results obtained from the in vitro assays performed on CS-based specimens, these demonstrated a marked antibacterial efficacy within 1–2 h of exposure to the bacterial inoculum. A mean reduction value of 3.57 log_10_ CFUs/cm^2^ was observed, with minimum and maximum values of R = 2.47 and 4.36 log_10_ CFUs/cm^2^, respectively. The CS/ZrPCHX specimens exhibited bactericidal activity against four out of the five bacterial strains tested, except for MRSA, which exhibited a lower reduction value of 2.47 log_10_ CFUs/cm^2^, indicating reduced susceptibility to the CS/ZrPCHX coating. These findings highlight the ability of both coatings to exert a pronounced and rapid antibacterial effect against all tested bacterial strains.

In this study, in addition to the two reference bacterial strains indicated in ISO 22196:2011, three bacterial strains—methicillin-resistant Staphylococcus aureus (MRSA, ATCC^®^ 43300), *Klebsiella pneumoniae* (ATCC^®^ BAA-1705), and Acinetobacter baumannii (ATCC^®^ 19606)—belonging to the ESKAPE pathogens were tested [46]. The term ESKAPE is an acronym that identifies six clinically relevant pathogenic bacteria (*Enterococcus faecium*, *Staphylococcus aureus*, *Klebsiella pneumoniae*, *Acinetobacter baumannii*, *Pseudomonas aeruginosa*, and *Enterobacter* spp.), responsible for severe nosocomial infections and known for their multidrug resistance. These pathogens are particularly problematic in hospital settings and represent a growing threat to global public health. A recent study updated the WHO Bacterial Priority Pathogens List (BPPL) of 2024 using a multicriteria approach to classify 24 antibiotic-resistant pathogens. These pathogens were ranked into three priority levels: critical, high, and medium. Among those with the highest scores were carbapenem-resistant *Klebsiella pneumoniae*, *Escherichia coli*, and *Acinetobacter baumannii*, as well as methicillin-resistant *Staphylococcus aureus* (MRSA). The inclusion of MRSA, *K. pneumoniae*, and *A. baumannii* in this study is highly relevant, as these bacteria belong to the ESKAPE group and are considered critical priorities by the WHO [47].

The results of this study highlight the potential of polycaprolactone-based samples (PCL/ZrPCHX) as fast-acting antibacterial coatings, capable of exerting a significant bactericidal effect within one hour against all tested bacterial strains, including those belonging to the ESKAPE group (MRSA, *Escherichia coli*, *Klebsiella pneumoniae*, and *Acinetobacter baumannii*), which are particularly relevant in healthcare and social care settings due to their important role in healthcare-associated infections. Moreover, the presence of average reduction values > 3.82 log_10_CFUs/cm^2^ indicates consistent bactericidal efficacy, albeit with slight variability among the tested strains. The data obtained suggest that this antibacterial coating can exert rapid and robust antimicrobial action, compatible with the requirements of applications in environments with a high infection risk.

Our data also confirms the potent antibacterial activity of the CS/ZrPCHX coating, demonstrating its ability to significantly reduce microbial load within a short exposure time (1 h), although limited to two Gram-negative strains, *Klebsiella pneumoniae* and *Acinetobacter baumannii*. Both are clinically significant pathogens frequently associated with healthcare-associated infections and known for their multidrug resistance. This coating demonstrated a marked antibacterial efficacy against the remaining strains within only 2 h of exposure to the bacterial inoculum.

The antibacterial activity of this CS-based coating was significantly faster and more effective against Gram-negative compared to Gram-positive bacteria, in agreement with numerous previous reports [48]. This difference is commonly attributed to structural variations in the cell envelopes of Gram-negative and Gram-positive bacteria. As is well known, Gram-positive bacteria possess a thick peptidoglycan layer in their cell wall, which acts as a protective barrier and may contribute to their increased resistance to antimicrobial agents [49]. In contrast, Gram-negative bacteria exhibit a more complex and dynamic cell membrane architecture, which has been associated with greater susceptibility to environmental stressors and antimicrobial treatments [50]. Several studies support this hypothesis, particularly highlighting that *Staphylococcus aureus* tends to be more resilient under adverse conditions and more resistant to a wide range of disinfectants compared to Gram-negative bacteria [51]. The coating’s performance against these strains suggests its high potential as a targeted intervention for infection control in high-risk settings, especially critical for high-touch areas, which are known hotspots for microbial transmission.

## 4. Conclusions

This study demonstrates the successful development of CS- and PCL-based coatings incorporating ZrPCHX particles, highlighting their promising potential for antimicrobial surface protection in healthcare settings.

The combined XRPD, FT-IR, and SEM characterization of ZrPCHX particles provides coherent evidence for the successful formation of the hybrid material while preserving the structural integrity of the zirconium hydrogen phosphate framework.

The wide characterization of the CS-based and PCL-based active coatings highlights the following results: Morphological analysis confirmed the homogeneous distribution of the active compound within both polymer matrices. Contact angle measurements showed that the surface wettability can be effectively tuned by the nature of the polymer matrix and the presence of antimicrobial particles: while CS-based coatings generally increased hydrophobicity, the incorporation of ZrPCHX enhanced hydrophilicity, potentially favouring better interaction with aqueous environments and microbial cells. FTIR analysis demonstrated that the addition of ZrPCHX does not alter the chemical structure of the polymer backbones, preserving the intrinsic properties of both CS and PCL. Adhesion tests confirmed excellent coating–substrate (PP) interactions, indicating an essential characteristic for real applications where prolonged exposure to moisture, mechanical stress, and cleaning agents is expected.

On the other hand, removability tests indicated different behaviours between the two systems, with CS-based coatings being gradually removable through abrasion and PCL-based coatings showing detachment once failure was initiated, highlighting an important consideration for future application-specific design. Cytocompatibility tests confirmed the safety of both of the developed coatings. In particular, PCL-based materials provide a more inert environment for fibroblasts, highlighting their good cytocompatibility and reinforcing their potential suitability for biomedical applications involving both direct and indirect contact with fibroblasts.

In terms of antibacterial performance, the coatings showed very interesting results. PCL/ZrPCHX coatings were particularly effective, displaying a rapid and strong bactericidal effect within just one hour against all tested strains, including multidrug-resistant bacteria from the ESKAPE group. CS/ZrPCHX coatings also demonstrated significant antibacterial activity, particularly against Gram-negative strains such as Klebsiella pneumoniae and Acinetobacter baumannii, which are among the most challenging pathogens in clinical environments. Although slightly slower in action, CS-based coatings achieved notable reductions in bacterial load within two hours, supporting their potential as antimicrobial surfaces.

The findings of this study offer a meaningful contribution to the ongoing efforts to prevent healthcare-associated infections (HAIs) and limit the spread of antibiotic-resistant bacteria. The demonstrated efficacy of these newly developed materials highlights their potential as protective coatings against multidrug-resistant pathogens. Moreover, the in vitro evaluation of bactericidal activity, conducted at different contact times with bacterial inoculum, provided valuable insights into the speed of the antimicrobial action. This parameter represents a crucial factor, as the timeliness of the response is decisive in ensuring effective infection control.

## Figures and Tables

**Table 1:** Mean reduction values (R) of bacterial colonies of all tested bacterial strains, following exposure to PCL/ZrPCHX and CS/ZrPCHX specimens, evaluated at different exposure times (*).

Bacterial Strains	PCL/ZrPCHX	CS/ZrPCHX
*S. aureus*, ATCC^®^ 6538P	R = 4.02 (1 h)	R = 3.88 (2 h)
MRSA, ATCC^®^ 43300	R = 3.82 (1 h)	R = 2.47 (2 h)
*E. coli*, ATCC^®^ 8739	R = 5.00 (1 h)	R = 4.21 (2 h)
*K. pneumoniae*, ATCC^®^ BAA-1705	R = 4.98 (1 h)	R = 2.93 (1 h)
*A. baumanii*, ATCC^®^ 19606	R = 4.60 (1 h)	R = 4.36 (1 h)

(*) Activity was tested by using ISO 22196:2011.

## Data Availability

Data is available from the authors upon request.
